# The Emerging Role of NK Cells in Immune Checkpoint Blockade

**DOI:** 10.3390/cancers14236005

**Published:** 2022-12-06

**Authors:** Alejandra Martinez-Perez, Candelaria Aguilar-Garcia, Segundo Gonzalez

**Affiliations:** 1 Department of Functional Biology, Immunology, Universidad de Oviedo, 33006 Oviedo, Spain; 2 Instituto Universitario de Oncología del Principado de Asturias (IUOPA), 33006 Oviedo, Spain; 3 Instituto de Investigación Sanitaria del Principado de Asturias (ISPA), 33011 Oviedo, Spain

## 1. Introduction

Natural killer (NK) cells are innate cytotoxic immune cells that play a fundamental role in anti-tumor immunity, particularly in hematological cancers, disseminated cancers, and metastasis [1,2,3]. Unlike T cells, NK cells lack the expression of antigen-specific receptors, instead expressing an array of activating and inhibitory receptors that bind to ligands whose expression changes upon cancer transformation. Activating receptors, including Natural Killer Group 2D (NKG2D), DNAX Accessory Molecule-1 (DNAM-1), and Natural Cytotoxicity Receptors (NCRs), recognize inducible ligands up-regulated in cancer cells (induced self-recognition) [4]. Inhibitory receptors, such as killing inhibitory receptors (KIRs) or CD94-NKG2A, mainly sense the loss of Human Leucocyte Antigen (HLA) class I molecules, a feature frequently observed in cancer cells (missing self-recognition). NK cells from patients with cancer also express a plethora of inhibitory immune checkpoints, including Programmed cell Death protein 1 (PD-1), Lymphocyte Activation Gene 3 (LAG-3), T cell immunoglobulin and mucin-domain containing-3 (TIM-3), T cell immunoreceptor with Ig, and ITIM domains (TIGIT) and B- and T-lymphocyte attenuator (BTLA) [5,6,7]. The balance between signals provided by activating and inhibitory receptors determines NK cell activation, which results in tumor cell killing and the release of cytokines, such as IFN-γ, that regulate the adaptive and innate anti-tumor immunity. A central role of NK cells in anti-tumor immunity is highlighted by several studies showing that high NK cell tumor infiltration and/or high levels of expression of activating receptors on this immune cell subset are associated with better prognosis and, contrarily, that NK cell dysfunction in the tumor microenvironment is associated with adverse clinical outcomes in multiple cancers [1,2,6,7,8,9]. Mechanistically, impaired NK cell function in cancer has been associated with a myriad of mechanisms, including the up-regulation of PD-1 [10]. PD-1 is an inhibitory co-receptor transiently expressed on activated T cells, B cells, and myeloid immune cells that specifically binds to programmed death-ligand 1 (PD-L1) and programmed death-ligand 2 (PD-L2) [11]. PD-L1 is constitutively expressed in a wide range of healthy cells, whereas PD-L2 is expressed in professional antigen-presenting cells during inflammation. Physiologically, PD-1 plays a central role in preventing autoimmunity. In advanced cancers, chronic T cell stimulation induces the up-regulation of the expression of inhibitory immune checkpoints, including PD-1, leading to an exhausted phenotype characterized by decreased T cell proliferation, functionality, and survival, thus hindering the anti-tumor immunity [12]. Immune checkpoint blockade (ICB), which interferes with negative signals provided by these molecules, has revolutionized cancer therapy, becoming the frontline therapy for many cancers, and, in certain cancers, such as melanoma, PD-1 inhibitors have largely replaced chemotherapy [13].

### The Manuscript by Quatrini L. et al. Published in Cancers

The manuscript by Quatrini L. and coauthors [14] brings to light the emerging role of PD-1 in NK cells and their contribution to the clinical success of anti-PD-1 therapy. While it was initially thought that blocking the PD-1/PD-L1 axis would only unleash T cell responses, mounting evidence shows that NK cells also make a relevant contribution to the clinical success of ICB-based therapy. PD-1 is not expressed on NK cells in most healthy individuals, but Quatrini L. et al. [14] convincingly review the evidence, showing that PD-1 expression is induced in peripheral and tumor-infiltrating NK cells in multiple cancers, including multiple myeloma, renal cell carcinoma, Kaposi sarcoma, digestive cancers, ovarian cancer, non-small-cell lung carcinoma (NSCLC), Hodgkin lymphoma, and others [10]. PD-1 pathway is an important determinant of the outcome of T cell response and mounting data suggest that it plays a similar role in NK cells. The inhibitory role of PD-1 in NK cells is highlighted by its correlation with intra-tumoral NK cell dysfunction and poor prognosis of patients in several cancers [9,10,15]. In this scenario, the contribution of NK cells to immunotherapy mediated by PD-1/PD-L1 blockade has convincingly been shown in transplantable, spontaneous, or genetically induced mouse tumor models [16]. Recent clinical evidence also suggests a relevant role of NK cells in the clinical effectiveness of PD-1/PD-L1 therapy, particularly in HLA class I deficient tumors [2]. Downregulation of HLA class I is a mechanism frequently developed by certain cancers, such as Hodgkin’s lymphoma, to avoid T cell recognition; however, these cancer cells are more efficiently identified and eliminated by NK cells (missing-self recognition) [16]. It is worth mentioning that myeloid leukemia cells induce PD-L1 expression on NK cells and their treatment with an anti-PD-L1 antibody results in NK cell activation and tumor regression [17]. This finding is highlighted by Quatrini L. et al. [14] because it provides a potential explanation as to why some patients with cancer lacking PD-L1 expression on cancer cells respond to the anti-PD-1/PD-L1 therapy.

Even though interferons and other inflammatory stimuli are well-known key determinants of the regulation of PD-1/PD-L1 expression, Quatrini L. et al. [14] remark the less-renowned role of glucocorticoids as an indispensable stimulus required for PD-1 surface expression on murine and human NK cells. Glucocorticoids alone are not sufficient for PD-1 induction in NK cells, and cytokines present in the tumor microenvironment, including IL-12, IL-15, and IL-18, are fundamental for PD-1 expression in human NK cells [18]. This finding has crucial clinical implications as synthetic glucocorticoids are frequently administered to cancer patients treated with chemotherapy and/or immunotherapy, and they may have a role in anti-PD1/PD-L1 therapy resistance.

Although ICB has been a revolution in cancer therapy, the rate of response across solid tumors is around 20% [19]. This means that a majority of patients with cancer do not benefit from ICB. Quatrini L. et al. [14] highlight two major avenues to improve the effectiveness of this therapy, namely combination therapy and the development of predictive biomarkers. On one hand, the co-expression of inhibitory receptors on NK cells and T cells, including NKG2A, TIM-3, LAG-3, and TIGIT, suggests a promising strategy to improve ICB by simultaneously targeting multiple immune checkpoints [5]. On the other hand, an unmet need in anti-PD-1/PD-L1 therapy is the identification of novel predictive biomarkers to better select those patients that may obtain the maximum benefit from this therapy [20]. The most known and used biomarker is PD-L1 expression levels, which are required for the administration of the anti-PD-1 antibody pembrolizumab in NSCLC, head and neck squamous cell carcinoma (HNSCC), urothelial carcinoma, esophageal carcinoma, gastric carcinoma, and cervical carcinoma, and for the administration of the anti-PD-L1 antibody atezolizumab in triple-negative breast carcinoma (TNBC) and urothelial carcinoma. However, as mentioned above, PD-1/PD-L1 blockade exhibits clinical effectiveness in some patients with cancer regardless of PD-L1 expression on tumor cells, thus suggesting a significant contribution of other immune cells, such as NK cells, to the success of this therapy [17]. Mismatch repair (MMR) deficiency is another biomarker of response to anti-PD-1/PD-L1 therapy, as these patients carry genomic instability with high mutational burden and abundant neoantigens, which render tumors more immunogenic for T cells [20]. A high mutational burden is itself associated with the response to immunotherapy [21]. No biomarkers related to NK cells have been identified yet. Given the emerging role of NK cells in the effectiveness of PD-1/PD-L1 blockade, Quatrini L. et al. [11] consider that it is foreseeable that the precise definition of the number, phenotype, or functionality of NK cells in the tumor microenvironment could be a novel predictive biomarker that might help to predict the response to ICB.

Finally, the manuscript by Quatrini L. et al. [14] remarks on the potential role of the soluble form of PD-1 (sPD1) in the modulation of anti-tumor immunity and its impact on tumor progression. PD-1 not only exists as a membrane protein but also as a soluble form that is generated, at least, by alternative splicing [22]. sPD-1 competes with membrane PD-1 for binding with its ligands, exerting important immune modulatory functions, impacting the survival of patients with cancer, and modulating the efficacy of the anti-PD-1 therapy [23]. Hence, sPD-1 could be a potential biomarker that should be evaluated in future preclinical and clinical studies. Further, a potential role of sPD-1 in NK cell biology remains to be established.

To conclude, Quatrini L. et al. [14] reviewed the role of PD-1 in NK cells and shed light on the similarities and differences between the biology of PD-1 in NK cells and T cells. A better understanding of the role of PD-1 will help to fully harness the anti-tumor potential of NK cells [2,3,24].
